# Barriers to vaccination in immunocompromised children: A needs assessment in children with childhood-onset SLE and inflammatory bowel disease

**DOI:** 10.3389/fped.2023.1103096

**Published:** 2023-03-01

**Authors:** Audrey R. Lloyd, Monica I. Ardura, Kelly Wise, Daniel J. Chavarin, Brendan Boyle, Vidya Sivaraman

**Affiliations:** ^1^ Internal Medicine, Division of Infectious Diseases, University of Alabama, Birmingham, AL, United States; ^2^ Pediatrics, Divisions of Infectious Disease, Nationwide Children’s Hospital and The Ohio State University, Columbus, OH, United States; ^3^ Pharmacy, Nationwide Children’s Hospital and The Ohio State University, Columbus, OH, United States; ^4^ Pediatrics, Rheumatology, Nationwide Children’s Hospital and The Ohio State University, Columbus, OH, United States; ^5^ Internal Medicine, Division of Internal Medicine, University of New Mexico, Albuquerque, NM, United States; ^6^ Pediatrics, Gastroenterology, Nationwide Children’s Hospital and The Ohio State University, Columbus, OH, United States

**Keywords:** vaccination, immunocompromised, children, rheumatic diseases, prevention, vaccine hesitancy

## Abstract

**Background:**

Vaccination of immunocompromised children (ICC) remains suboptimal.

**Methods:**

Needs assessment surveys were administered to patients and caregivers during routine ambulatory visits to the rheumatology and gastroenterology clinics at Nationwide Children's Hospital (NCH) from January 1 through August 31, 2018, and to community primary care physicians (PCPs) at their monthly meeting and electronically.

**Results:**

Completed surveys were received for 57 patients **(**31 with childhood-onset systemic lupus erythematosus (c-SLE) and 26 with inflammatory bowel disease (IBD)) and 30 PCPs. Of the patient cohort, 93% (*n* = 53) felt their PCP was well informed about vaccines and 84% (*n* = 47) received vaccinations from either their PCP or local health department. Two patient surveys noted concerns of vaccine safety. Among the 30 responses completed by PCPs 50% (*n* = 15) preferred to provide all vaccines themselves, however, only 40% (*n* = 12) of PCPs felt “very confident” when providing vaccines to ICC. Further, 83% (*n* = 25) did not stock the 23-valent pneumococcal vaccine and only 27% (*n* = 8) routinely recommended vaccination of household contacts.

**Conclusions:**

Our study found a discordance between parent and PCP comfort in vaccinating ICC, highlighting an important barrier to vaccination in this patient population. In our cohort of patients, vaccine hesitancy was not a barrier to vaccination.

## Background

Immunocompromised children (ICC) are at increased risk for vaccine preventable infections and complications from these infections (1–4). However, for unclear reasons vaccination of these high-risk patients remains suboptimal (5, 6). In the United States, vaccination in ICC can be provided by subspecialists, PCPs or local health departments. While subspecialists can obtain access to vaccines, administering them in a busy subspecialty clinic is challenging due to time and staffing limitations. The lack of centralized vaccine databases is another barrier to documenting vaccines given in different clinics. Most subspecialty clinics provide influenza vaccines, and some provide pneumococcal or hepatitis B vaccines. Other childhood vaccines are typically given in the PCP office or local Health Department.

At Nationwide Children's Hospital (NCH), review of vaccination records found that the majority of children with childhood-onset systemic lupus erythematosus (c-SLE) or inflammatory bowel disease (IBD) had not received the recommended age-appropriate vaccinations for their underlying disease or extent of immunosuppression. For example, less than 10% of patients with c-SLE seen between January 2015 to August 2016 had received the 23-valent pneumococcal (PPSV23) vaccine as recommended by the Center of Disease Control and Prevention (CDC). Our Rheumatology clinic began providing the PPSV23 as a quality improvement initiative resulting in increasing pneumococcal vaccination rates to over 90% in patients with c-SLE (7). In the Division of Gastroenterology only 30% of patients with IBD starting anti-TNF medications had evidence of hepatitis B immunity (8). This has prompted hepatitis B vaccine re-vaccination in the GI clinic to non-immune patients. To better identify barriers preventing vaccination in this patient population, we performed a needs assessment survey in these patients and their caregivers and to assess vaccine practices in community PCPs.

## Methods

We created and administered two needs assessment surveys: One was administered to patients with c-SLE or IBD receiving care at the rheumatology and gastroenterology clinics at NCH or their caregivers, between January 1 to August 31, 2018, as a convenience sample during routine visits for disease management. Surveys were completed by patients 18 years or older, or by the parent or caregiver for younger patients, with an ability to opt out if desired. We reviewed electronic medical records of the surveyed patients for demographics, medication exposure, and diagnosis. The second survey was distributed to community primary care providers (PCP) at their monthly meeting followed by an email survey to PCPs who were not present at the meeting. Responses were recorded in a Redcap® database for statistical analysis. Two sample test of proportions was used to determine significance between proportions. Analyses were performed using SAS 9.4. Questions on the survey are provided in Supplementary S1 and S2. This study was performed as an internal quality improvement project to improve processes of care and was deemed exempt from review by the Institutional Review Board.

## Results

**Figure 1:**
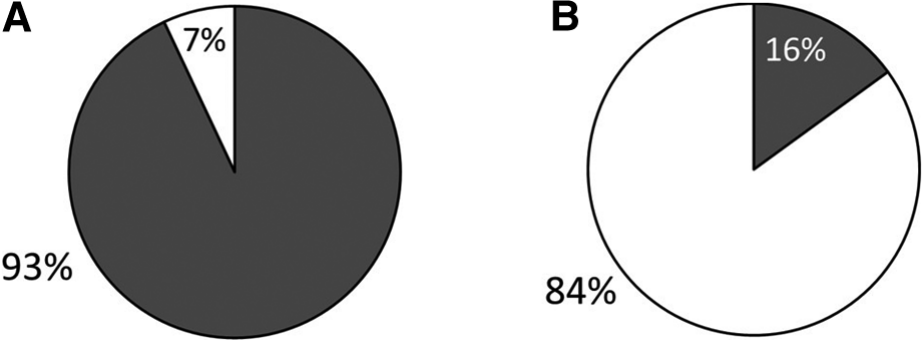
Caregiver-reported data on vaccination discussion and administration in IBD and c-SLE. Grey: Subspecialty clinic; White: PCP clinic or local health department. (**A**) Discussion of vaccinations in past year. (**B**) Administration of vaccines in the past year.

**Figure 2:**
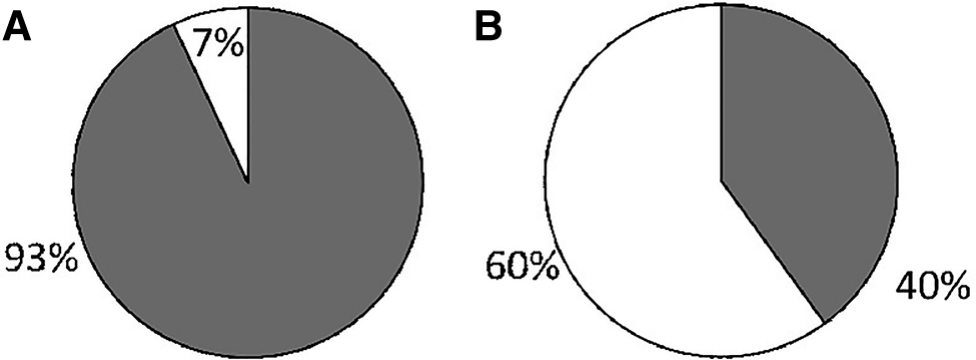
Caregiver-reported impression of PCP's knowledge of vaccination in ICC compared to PCP-reported confidence when providing vaccines to ICC. Grey: Well informed; White: Not well informed. 2A: Caregiver report. 2B: PCP report.

We received surveys for 57 patients: 31 with c-SLE and 26 with IBD, with a response rate of 100%. Patient characteristics are detailed in Table 1. Of the 26 patients with IBD, 22 (85%) had Crohn's disease (CD) and 4 (15%) had ulcerative colitis (UC). The majority of c-SLE were non-white and female while IBD patients were mostly white with sexes represented evenly. Both patient categories had a median age of 16 years (Table 1). Of all patients surveyed, 44 (77%) were receiving disease modifying anti-rheumatic drugs (DMARDs), 32 (18%) were receiving systemic corticosteroids, and 13 (23%) were receiving biologic drugs. Fifty-three patients (93%) reported their subspecialist discussed vaccines in the past year, most commonly influenza, human papilloma virus, pneumococcal and hepatitis B vaccines (Figure 1A). Despite discussing vaccines with their subspecialist, the majority of patients (*n* = 47, 84%) received their vaccines from their PCP or Health Department and 8 (16%) patients reported they received their vaccines from their subspecialist (Figure 1B). The proportion of patients reporting that their subspecialists had discussed vaccines (53/57; 92.9%) was significantly different from the proportion receiving vaccines from their subspecialists (9/56; 16%, *p* < 0.001). There was one missing response for vaccine administration. The remaining 84% of patients received vaccines from their PCP or local health department. Fifty-three patients (93%) reported they thought their PCP was well-informed about vaccines (Figure 2A). Only 2 (4%) patients or caregivers reported concerns for possible vaccine adverse effects, including disease flare triggered by a vaccine. Fifty-five (96%) of respondents reported that the patient was up to date with their vaccines by self-report. Vaccine safety was not cited as a concern among the 2 (4%) patient surveys in which parents self-reported that their children were not up to date with vaccines. Since there is no fully centralized database for vaccinations in Ohio and patients from neighboring states are also seen in our clinics, we were unable to confirm patient vaccination status.

**Table 1:** Patient demographics.

	SLE (*n* = 31)	IBD(*n* = 26)	All subjects (*n* = 57)
**Demographic data**			
Age: Median (Range)	16 (8–21)	16 (4–22)	16 (4–22)
Caucasian (%)	11 (35)	21 (81)	32 (56)
Other Race (%)	20 (65)	5 (19)	25 (44)
Female Gender (%)	28 (90)	13 (50)	41 (72)
**Medications**			
Daily Systemic Steroids	18 (58)	0 (0)	18 (32)
Non-Biologic DMARDs	31 (100)	14 (54)	45 (78.9)
Biologic DMARDs	4 (13)	9 (35)	13 (23)

We received 30 survey responses from community PCPs, with an estimated response rate of >90% for in-person survey and less than 10% for electronic survey. Twenty-one (70%) respondents reported over 20 years’ experience in practice and 15 (50%) preferred to provide all vaccines to their ICC patients. The majority of PCPs (73.3%) reported seeing 1–20 children with rheumatic disease or IBD per year in their practice, while 26.7% reported seeing 21–49. Of note, only 40% of PCPs stated they felt “very confident” about providing vaccines to ICC (Figure 2B) in contrast to 93% of patients reporting that their PCP was well-informed regarding indicated vaccines, with a *p*-value of <0.001. Practitioners cited uncertainty about patient's immunosuppressive medications and concern for exacerbating the underlying illness as the main reasons for their lack of confidence in providing vaccines to ICC. Finally, most PCPs, 14 of 16 (87.5%), stated they did not stock the PPSV23 in their office.

**Figure 3:**
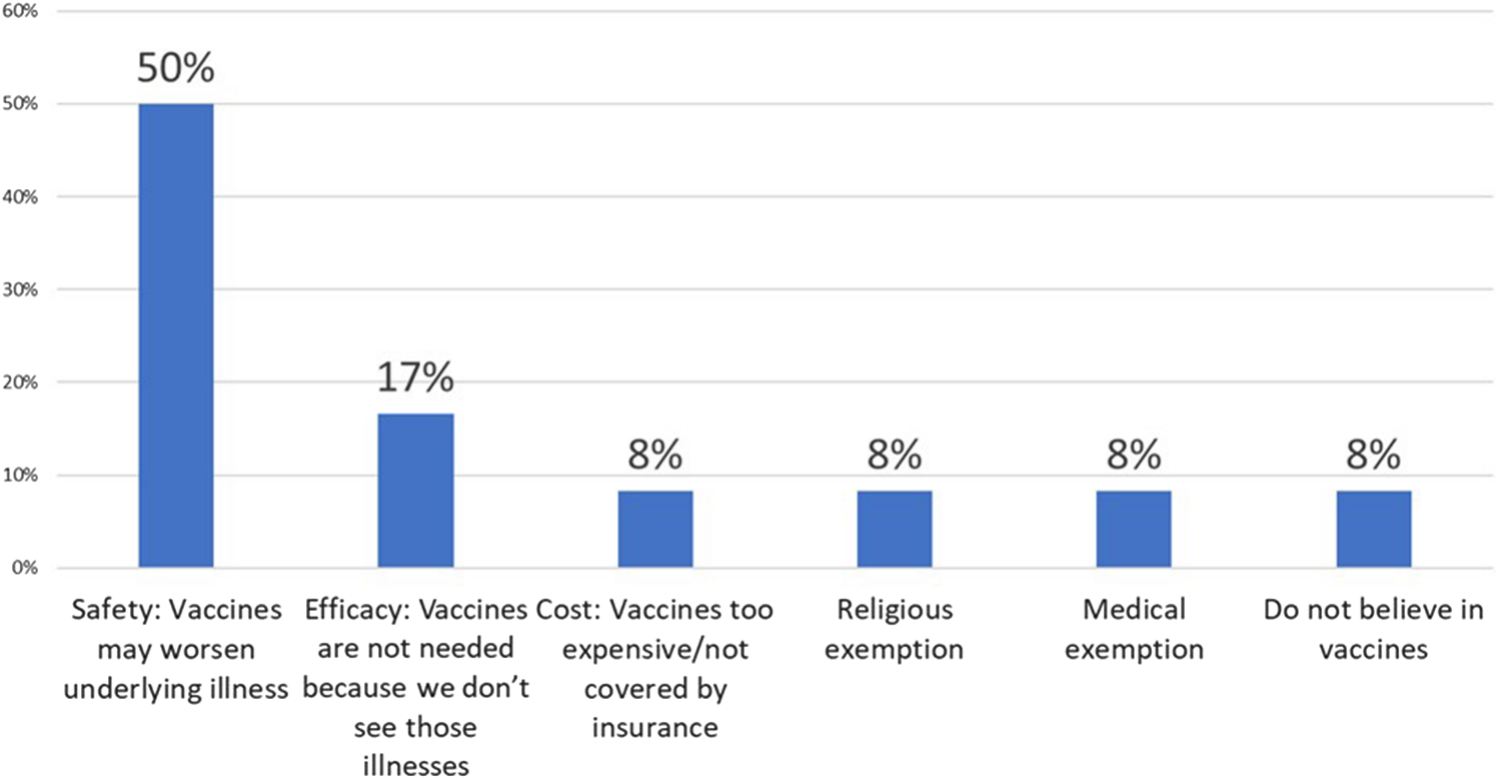
Reasons for vaccine refusal in ICC per PCP. Other: 1% “Anti-vaxer philosophy”.

When vaccinating ICC, only 40% of PCPs recommended vaccination of household contacts of ICC greater than 50% of the time. Additional comments added by 6 PCPs (22%) included “100% with flu” to “Never- Am I supposed to?” Two PCPs stated they recommended vaccination of household contacts only if the patient's specialist recommended it. Notably, similar to the patient survey results, PCPs did not report vaccine hesitancy as a major barrier to vaccination of ICC and their families. Eighty-five percent of PCPs reported no refusal or minimal vaccine refusal among ICC patients and parents. The most common reasons for vaccine refusal were concern that the vaccine may worsen the underlying illness or trigger a disease flare, followed by lack of necessity due to rarity of infection, cost, religious or medical exemptions and one report of “anti-vaxer philosophy”. Reasons for vaccine refusal in ICC as reported by PCPs are outlined in Figure 3. When asked to identify strategies to improve vaccination of ICC in their clinics, PCPs asked for better documentation and clear recommendations from specialists regarding the vaccination of ICC patients and their household contacts.

## Conclusion(s)

ICC are at increased risk for vaccine preventable diseases (1, 2). Despite guidelines from the CDC and American Academy of Pediatrics (AAP) recommending vaccination of ICC and their household contacts, rates of vaccination among patients with c-SLE or IBD at our institution remain suboptimal (7, 8)*.* In our survey of patients and PCPs, there was discordance between patients reporting a perceived strong confidence and trust in their PCP, and PCPs who report lack of comfort in vaccinating ICC. Despite AAP, CDC, and European Alliance of Associations for Rheumatology (EULAR) recommendations (1, 2, 9) to vaccinate ICC and strong evidence that inactivated vaccines are safe and do not increase risk of disease flare (10), PCPs and patients still cited concerns for triggering a disease flare and concerns for vaccine safety in ICC (11, 12). This gap represents an opportunity for improved education among PCPs regarding the vaccination of ICC and communication with subspecialty physicians.

While 50% of PCPs stated they preferred to provide all vaccinations to ICC themselves, 60% reported lack of comfort in vaccinating this population and the majority of the offices surveyed did not carry the PPSV23, a vaccine that is specifically indicated in many high-risk groups including children receiving immunosuppressive therapy. The majority of PCPs (73.3%) reported seeing only 1–20 children with rheumatic disease or IBD and 26.7% saw 21–49 children with these diagnoses each year in their practice. These numbers could reflect truly lower numbers of patients with these conditions in their practice or possible lack of awareness of a patient's underlying diagnoses and immunocompromised status at the time of vaccination.

We do not expect that the focused quality improvement initiatives around pneumococcal and hepatitis B vaccination would have impacted the confidence of PCPs in providing vaccines to ICC to a significant extent since they did not include an educational component for PCPs. However, they may have improved awareness of need for certain vaccines in this population. Since the PCPs who completed the survey were not matched to the patients and caregivers, we are unable to confirm a direct impact of our institutional vaccination efforts on the comfort of PCPs in vaccinating ICCs.

The majority of PCPs surveyed (60%) reported that they did not routinely recommend vaccination of household members. In fact, written comments showed that several PCP were unaware of current guidelines regarding the vaccination of household contacts of ICC. Patient and parent vaccine hesitancy was not reported as a major barrier to vaccination among patients or PCPs. Previous literature by Lawson et al. (4), showed that increased discussion of vaccinations in the subspecialist's office led to increased vaccination rates in ICC and decreased vaccine hesitancy. Our survey showed a high rate of vaccine discussion during subspecialist visits (93%), which is encouraging and can be utilized for future improvement.

Concurrent with this project and others at NCH, our rheumatology clinic has begun providing the PPSV23 and a quality improvement initiative has resulted in increasing pneumococcal vaccination rates to over 90% in patients with c-SLE (7). Therefore, we elected to survey these patients and their caregivers to assess their perceptions related to vaccination. In the gastroenterology clinic, only 30% of patients with IBD starting anti-TNF medications had evidence of hepatitis B immunity (8). This has prompted hepatitis B vaccine re-vaccination in the gastroenterology clinic to non-immune patients. We would like to expand this survey to caregivers of children with JIA and other autoimmune and rheumatic diseases in the future.

This study has several important limitations: this was a single center study with a small sample size of 30 PCPs and 57 patients who were selected as a convenience sample. The majority PCPs who responded to the survey reported having limited exposure to ICC, which could contribute to lower level of comfort and knowledge of vaccine recommendations for this population. The PCPs were not matched to the patients/caregivers completing the surveys; hence we are not able to comment on the confidence of PCPs with direct experience with ICC with those who did not. While the number of ICC seen by the PCPs was lower than expected the need for awareness of specific vaccination guidelines for this vulnerable population remains vital. Our survey results highlight the need for increasing awareness of specific vaccination requirements for this population as reflected by comments from several PCPs asking for guidelines, algorithms or detailed communication from the subspecialist on required vaccines through clinic notes or letters.

The study was performed before the COVID-19 pandemic and rates of vaccine hesitancy may have changed since it was completed (13, 14). Lastly, patient/parent-reported vaccination status may have been inaccurate. Notably, we were unable to confirm patients' vaccination status since we do not have a complete centralized vaccination database.

In the future, we would like to expand this survey to include more PCPs and pediatric centers and assess changes in vaccine hesitancy after the COVID-19 pandemic. We also plan to match patient responses with those of their PCPs, to see if PCP answers change with exposure to ICC. Our survey highlights that the responsibility for vaccinating ICC is a shared responsibility between PCP and pediatric subspecialists (2, 15). Improving communication between subspecialists and PCP by providing PCP with clear guidelines on vaccination of ICC and their household contacts is another area for future improvement. Vaccinations are an important part of keeping ICC safe. Even with clear national guidelines, access to recommended vaccines and lack of education about appropriate vaccination in ICC among patients and health care providers remain significant barriers and areas for improvement.

## Data Availability

The raw data supporting the conclusions of this article will be made available by the authors, without undue reservation.
