# Supplementary material for: Barriers to vaccination in immunocompromised children: A needs assessment in children with childhood-onset SLE and inflammatory bowel disease
Source: Front Pediatr. 2023 Mar 1;11:1103096. doi: 10.3389/fped.2023.1103096 (PMC10014617; doi:10.3389/fped.2023.1103096)
Supplement: Supplementary file 1 [file Datasheet1.docx]

Vaccination Survey:

Thank you for taking the time to complete this brief survey, which will take 2-3 minutes to complete. At Nationwide Children’s Rheumatology Clinic, we are working to improve our care of immunosuppressed patients. We need your feedback in order to improve our processes for vaccination in our patients to prevent infections.

Sincerely,

Rheumatology providers

1. What is the age of your child?
2. In the past year, has your child’s specialty provider discussed vaccinations with you?
   1. Yes
   2. No
3. If yes, which of the following vaccines were discussed:
   1. Flu
   2. MMR
   3. Varicella (Chicken pox)
   4. Pneumococcal (Pneumonia)
   5. Meningococcal (Meningitis)
   6. HPV
   7. Other
4. In the past year, has your provider discussed the following with you:
   1. Vaccines that are contraindicated due to your disease or medications
   2. Vaccines are recommended for your child due to your disease or medications
5. If vaccines were recommended, do you have any concerns regarding giving the recommended vaccines to your child?
   1. Yes
   2. No
6. If yes, what is the reason for your concern:
   1. Risk of adverse reaction to the vaccine
   2. Vaccine could trigger a disease flare
   3. Child may not respond due to immunosuppressive medications
   4. Other: Please specify
7. Where does your child usually receive vaccines?
   1. PCP office
   2. Health Department
   3. Subspecialist office

Continued ->

1. Does your PCP/ Health department feel well-informed about your child’s vaccine recommendations due to their disease or medications?
   1. Yes
   2. No
2. If no, which vaccines were not discussed/ recommended?
   1. Flu
   2. MMR
   3. Varicella (Chicken pox)
   4. Pneumococcal (Pneumonia)
   5. Meningococcal (Meningitis)
   6. HPV
   7. Other
3. Have you had any financial or insurance concerns associated with your child’s vaccinations?
   1. Yes
   2. No
4. My child is up to date on recommended vaccines:
   1. Yes
   2. No
   3. No, prefer not to vaccinate
5. What other barriers have your encountered in obtaining age-appropriate vaccinations for your child?
6. Please share with us additional comments or suggestions regarding vaccination for your provider
