# Supplementary material for: Barriers to vaccination in immunocompromised children: A needs assessment in children with childhood-onset SLE and inflammatory bowel disease
Source: Front Pediatr. 2023 Mar 1;11:1103096. doi: 10.3389/fped.2023.1103096 (PMC10014617; doi:10.3389/fped.2023.1103096)
Supplement: Supplementary file 2 [file Datasheet2.docx]

**OPTIMIZING PROTECTION AGAINST VACCINE PREVENTABLE INFECTIONS AMONG IMMUNOCOMPROMISED CHILDREN AND ADOLESCENTS**

National guidelines recommend that primary care providers (PCP) and pediatric specialists who care for **immunocompromised children** share responsibility in ensuring that appropriate vaccinations are administered to these patients. Despite this, there are opportunities for improvement in vaccine delivery to high risk children and adolescents. We are attempting to identify obstacles and possible solutions to improving the immunization process for these high risk patients receiving their medical or subspecialty care at Nationwide Children’s Hospital (NCH) and would appreciate your helpful feedback by completing this survey.

For this survey, immunocompromised child (ICC) refers to children with underlying chronic immune-mediated or autoimmune diseases receiving immunosuppressive medications (e.g., TNF-alpha inhibitors such as infliximab, adalimumab or other biologic medications, 6 mercaptopurine, methotrexate, systemic corticosteroids). “I” and “you” in the questions refer to the PCP.

1. How would you describe your practice setting:

- Private practice, small group (0-5 providers)
- Private practice, large group (>5 providers)
- Academic, affiliated with NCH
- Academic, not affiliated with NCH

1. How many years have you been in practice?
   - 0-5
   - 6-10
   - 11-20
   - >20
2. Do you routinely provide immunizations to patients in your office?
   - Yes
   - No
3. How many patients with underlying rheumatologic diseases do you estimate you take care of in your practice each year?

- 0
- 1-20
- 21-49
- >50
- Unsure

1. Please choose one statement that best applies to your practice style in evaluating all patients with a rheumatologic disease:
   - I do not routinely review their vaccination status
   - I routinely review their vaccination status
   - I routinely review their vaccination status and provide vaccination as appropriate, after contacting the subspecialist directly to review plans for immunosuppression.
   - I routinely review their vaccination status and provide vaccination as appropriate without previously contacting the subspecialist
   - I routinely review their vaccination status and refer the patients back to the subspecialist for vaccination, as appropriate
   - Other _________________
2. Please choose one statement that best applies to your practice style in vaccinating patients with an underlying rheumatologic odisease:
   - I prefer to provide all vaccines to these patients in my office
   - I prefer to provide some of the vaccines to the patients in the office
   - I prefer that vaccines for ICC be provided by the specialist (rheumatology, GI)
   - I have no preference of who provides vaccinations to the ICC
   - Other _________________
3. In general, I feel confident in providing vaccination to ICC in X % of my medical encounters:
4. ≤10%
5. 11-50%
6. 51-89%
7. ≥90%
8. What barriers do you face when immunizing ICC (number all choices that apply, in order of highest barrier =1 to lowest barrier = 9)
   - I do not have ready access to the patient’s immunization record to review
   - I don’t have the time to review the vaccine record and administer vaccine
   - I don’t feel well informed on which vaccines to provide to the ICC
   - I don’t feel well informed on the immunosuppressive medications and their impact on immunizations
   - I am concerned about exacerbating the chronic underlying illness
   - I don’t feel there is a good system for coordinating additional vaccine doses
   - I do not feel vaccination would be efficacious due to immunosuppression
   - Patient or parent refuses vaccination
   - Other _______________
9. The following vaccines are routinely assessed and provided for ICC in my practice (choose all that are applicable):

- Influenza, inactivated (IIV)
- Hepatitis B vaccine (HBV)
- Varicella vaccine (VZV)
- Pneumococcal, conjugate (PCV13)
- Pneumococcal, polysaccharide (PPSV23)
- Human papillomavirus (HPV)
- Meningococcal
- Tetanus, diphtheria, acellular pertussis (Tdap)
- Other: ___________________

1. My practice does not stock the following vaccines (choose all that are applicable):
   - Influenza, inactivated (IIV)
   - Hepatitis B vaccine (HBV)
   - Varicella vaccine (VZV)
   - Pneumococcal, conjugate (PCV13)
   - Pneumococcal, polysaccharide (PPSV23)
   - Human papillomavirus (HPV)
   - Meningococcal

- Tetanus, diphtheria, acellular pertussis (Tdap)
  - Other: _____________

1. Regarding evaluation of serologic response to vaccines in ICC (choose one, best answer):
   - I routinely assess for serologic response after immunization
     1. If yes, for which vaccines (choose all that are applicable):
        1. Pneumococcus
        2. Hepatitis B
        3. Diphtheria
        4. Tetanus
        5. Meningococcal
        6. Other: _________________
   - I do not routinely assess for serologic response after immunization
   - Other __________________
2. When vaccinating ICC, I frequently recommend immunization of household contacts:

- Each time I provide vaccination to the ICC
- <10% of time
- 11-50%
- >50%
- Depends (describe)_________________

10. Have you encountered immunocompromised patients/families who choose not to be vaccinated?

- Yes, but this is a small minority of ICC patients
  - Most frequent reason(s) for declining vaccination: _________________
    - Cost: Vaccines too expensive/not covered by insurance
    - Efficacy: Vaccines are not needed because we don’t see those illnesses
    - Efficacy: Vaccines may not be as robust so why bother
    - Safety: Vaccines may worsen underlying illness
    - Religious exemption
    - Medical exemption
    - Other __________________
- Yes, this is the majority of ICC patients
  - Most frequent reason(s) for declining vaccination:
    - Cost: Vaccines too expensive/not covered by insurance
    - Efficacy: Vaccines are not needed because we don’t see those illnesses
    - Efficacy: Vaccines may not be as robust so why bother
    - Safety: Vaccines may worsen underlying illness
    - Religious exemption
    - Medical exemption
    - Other __________________
- No
- Unsure

11. Please provide your thoughts on how to improve communication of vaccination strategies between PCP and specialists:

Thank you for your willingness to improve the care of patients at Nationwide Children’s Hospital!
